# Supplementary material for: scDiffusion: conditional generation of high-quality single-cell data using diffusion model
Source: Bioinformatics. 2024 Aug 22;40(9):btae518. doi: 10.1093/bioinformatics/btae518 (PMC11368386; doi:10.1093/bioinformatics/btae518)
Supplement: btae518_Supplementary_Data [file btae518_supplementary_data.pdf]

scDiffusion: conditional generation of high-quality single-cell data using diffusion model

Erpai Luo[1,#], Minsheng Hao[1,#], Lei Wei[1], Xuegong Zhang[1,2,*]

[1]MOE Key Lab of Bioinformatics and Bioinformatics Division of BNRIST,
Department of Automation, Tsinghua University, Beijing 100084, China
[2]School of Life Sciences and School of Medicine, Tsinghua University, Beijing 100084, China

Supplementary Materials

S1 Model training strategy and ablation study

Here, we showed the details of the model training strategy. In general, we first finetuned the pre-trained model and then trained the diffusion backbone using the finetuned autoencoder. After, we trained the conditional controller aside from the diffusion model and found the appropriate parameters to guide the conditional generation in the inference phase. The details are as follows.

First, we finetuned the autoencoder from the pre-trained weights. The parameters' tuning target is to minimize the training loss, which is the mean square error of the reconstructed scRNA-seq profile. According to the results shown in Table S1, we suggest the user to finetune the model from the pre-trained weights to accelerate the tuning process and improve the model's performance.

Next, we trained the diffusion backbone cooperated with the trained autoencoder. The parameters' tuning target is to optimize the evaluation metrics such as LISI and RF AUC. We conducted a series of ablation studies to evaluate different model and training settings (Table S4). Overall, the results indicated that the performance of scDiffusion is not strongly affected by these parameters. Besides, although increasing the number of parameters in the model can improve model performance, the time for training the model rises accordingly. Considering the balance between model performance and training cost, we used the current set of model parameters. We suggest the user to increase the number of model parameters or increase the diffusion steps if they have particularly high requirements for generation quality.

After, we trained the cell classifier according to the need of conditional generation. The parameters' tuning target is to optimize the classification accuracy. The hyperparameter γ is used to control the impact of the classifier when generating cells conditionally, which is only used in the inference stage. We examined how different γ values affect the conditional generation process when generating kidney cells in the Tabular Muris dataset (Table S5). The KNN AUC is the AUC score of the KNN model when distinguishing the generated kidney cells and the real kidney cells. The CellTypist Acc is the accuracy of CellTypist that trained on all the cell types of Tabular Muris. As the classifier weight γ increased, the CellTypist's accuracy to the kidney increased, while the AUC score of the KNN model became worse. We found a sharp increase of

[#] These authors contributed equally to this work.
[*] Corresponding Author. Email: zhangxg@tsinghua.edu.cn

the CellTypist Acc and a stable KNN AUC performance when γ was set to 2, which was also the default value in our model. We suggested the user use this default value in most cases.

For other training parameters, we would like to empirically give some reference parameter settings based on our experiments. The parameter w in Eq. 3 and Eq. 5 controls the generation variety, and we suggest to set it as the default value. The finetuning steps of the autoencoder were 50,000 in our experiments. Based on the experiments shown in Tab S1, we suggest the user to train the autoencoder with more than 150,000 steps to obtain a realistic generation result if training from scratch. The training steps of the diffusion backbone and classifier were 800,000 and 200,000 in our experiments, which is the hyperparameters for training a dataset with about 80,000 cells. We suggest the user adjust this parameter according to the size of their dataset.

S2 Supplemental Tables

Model	AE training steps	SCC	MMD	LISI	RF AUC
With finetuned foundation model	5e4	0.9804	0.0151	0.8763	0.7325
From scratch	5e4	0.9763	0.0322	0.8631	0.8862
From scratch	10e4	0.9760	0.0337	0.8630	0.8398
From scratch	15e4	0.9758	0.0401	0.8453	0.8251

Table S1: Performance of scDiffusion models with the finetuned foundation model or trained from scratch. AE training steps: the total training steps of the autoencoder.

Tissue	Control	scDiffusion	scDesign	cscGAN
Bladder	0.987	0.987	1.000	0.000
Heart and Aorta	0.961	0.665	1.000	0.000
Kidney	0.985	0.915	1.000	0.000
Limb Muscle	0.972	0.917	1.000	0.000
Liver	0.989	0.992	1.000	0.000
Lung	0.982	0.941	1.000	0.069
Mammary Gland	0.966	0.899	1.000	0.247
Marrow	0.987	0.953	0.998	0.016
Spleen	0.991	0.996	0.997	0.066
Thymus	0.956	0.925	1.000	0.090
Tongue	1.000	0.996	1.000	0.000
Trachea	0.998	0.983	1.000	0.001

Table S2: Classification accuracy between real cells and generated cells for different organs in the Tabular Muris dataset. Control: the test set of real cells; scDiffusion: scDiffusion-generated cells; scDesign: scDesign3-generated cells; cscGAN: cscGAN-generated cells. Cells generated by scDiffusion achieve similar accuracy compare to the control group.

Cell type	Control	scDiffusion	scDesign	cscGAN
CD14+ Monocyte	0.914	0.834	0.930	0.000
CD19+ B	0.748	0.902	0.993	0.000
CD34+	0.936	0.810	1.000	0.000
CD4+/CD25 T Reg	0.500	0.480	0.714	0.000
CD4+/CD45RA+/CD25- Naive T	0.238	0.000	0.622	0.000
CD4+/CD45RO+ Memory	0.264	0.188	0.651	0.000
CD56+ NK	0.886	0.944	0.971	0.000
CD8+ Cytotoxic T	0.682	0.772	0.775	0.003
CD8+/CD45RA+ Naive Cytotoxic	0.719	0.965	0.707	0.998
Dendritic	0.559	0.609	0.980	0.000

Table S3: Classification accuracy between real cells and generated cells for different cell types in the PBMC68k dataset. Control: the test set of real cells; scDiffusion: scDiffusion-generated cells; scDesign: scDesign3-generated cells; cscGAN: cscGAN-generated cells. Cells generated by scDiffusion achieve similar accuracy compare to the control group. CellTypist-generated cells showed lower accuracy in some cell types when training and validating under real data, indicating that different cell types in the PBMC68k are harder to distinguish than the organ types in Tabular Muris (Table S2). Since the target of scDiffusion is to preserve the distribution and properties of the original data as much as possible, the results of scDiffsuion-generated cells are aligned with the results of real cells, leading to poorer performance in some cell types.

Model	SCC	MMD	LISI	RF AUC	Training time (s)
Current setting	0.9872	0.0027	0.9051	0.6652	19.4
5-layer MLP	0.9875	0.0025	0.9061	0.6524	24.6
3-layer MLP	0.9871	0.0061	0.8772	0.7088	17.2
Wider MLP	0.9872	0.0022	0.9087	0.6523	21.0
Narrower MLP	0.9870	0.0075	0.8515	0.7258	18.1
Double Lr	0.9872	0.0028	0.9034	0.6683	–
one-half Lr	0.9872	0.0036	0.9040	0.6679	–
Double steps	0.9910	0.0024	0.9014	0.6481	38.8

Table S4: Ablation study of scDiffusion. Training time: the time for training a complete diffusion process. Lr: learning rate.

Hyperparameter γ	KNN AUC	CellTypist Acc
0.5	0.5117	0.22
1	0.5050	0.588
2	0.508	0.918
4	0.5619	0.991
8	0.5786	1.0

Table S5: The results of conditional generated Kidney cells of the Tabular Muris dataset under different classifier control weights γ.

S3 Supplemental Figures

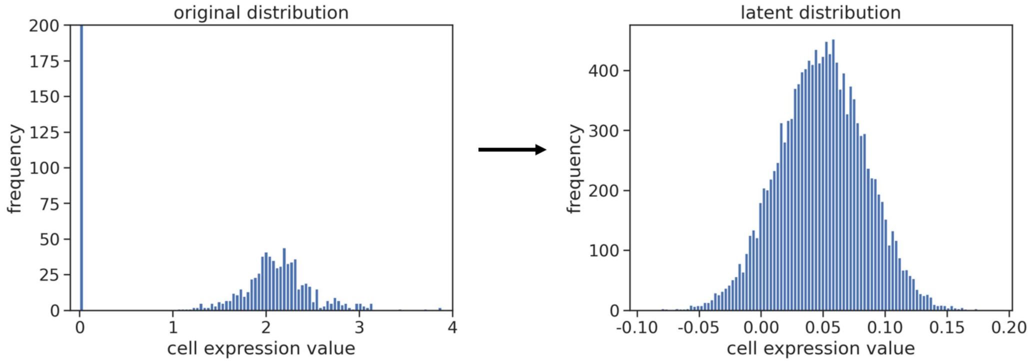

Figure S1: Distribution of original gene expression and latent embeddings derived by the autoencoder.

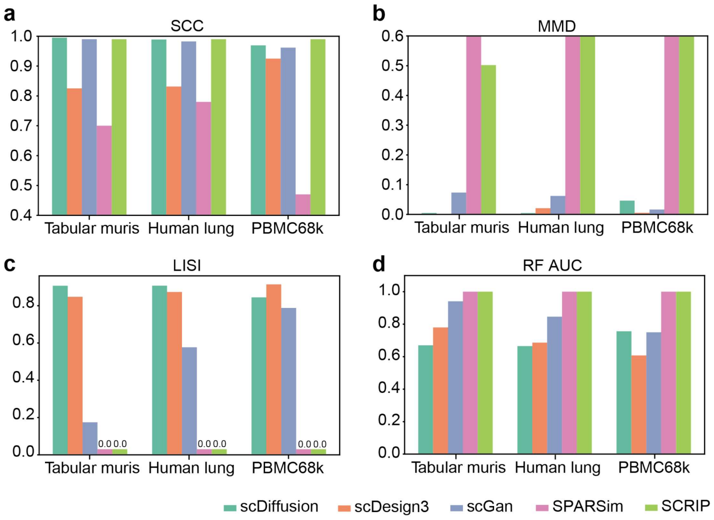

Figure S2: The performance of different methods for generating cells, evaluated by different metrics.

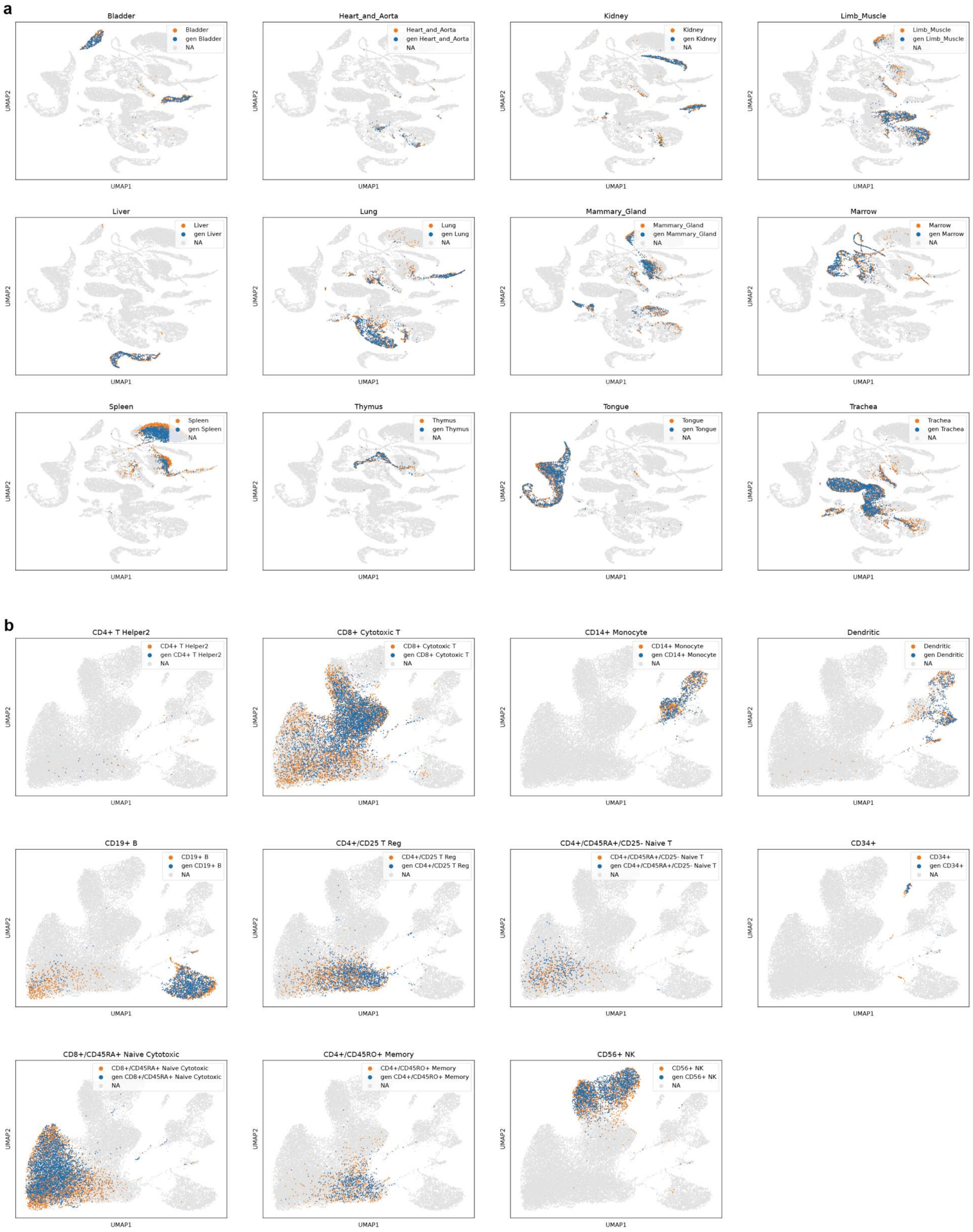

Figure S3: UMAP of conditionally generated cells. (a) The Tabular Muris dataset. (b) The PBMC68k dataset.

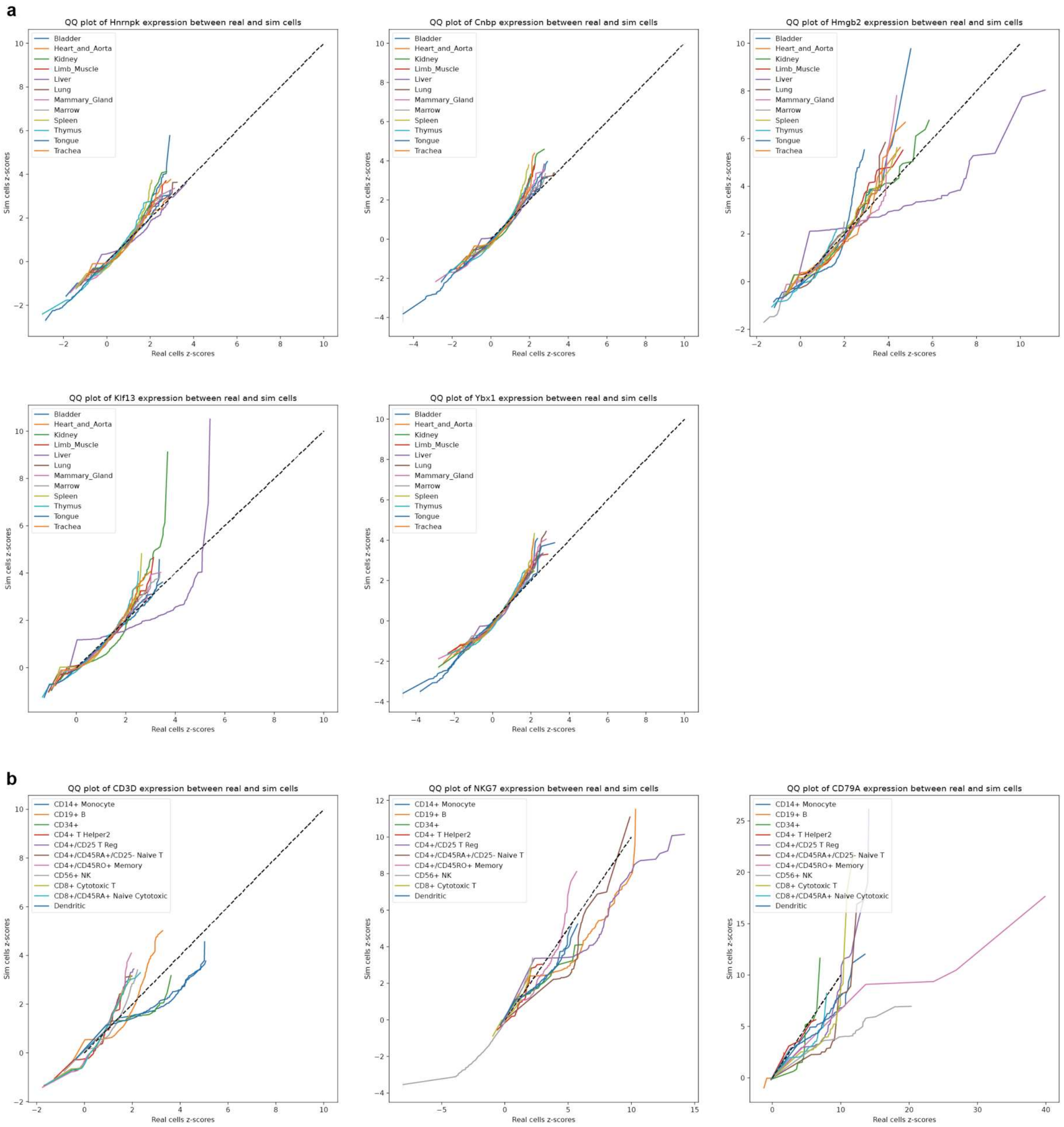

Figure S4: QQ-plots of expression of feature genes in the real and generated data. (a) The Tabular Muris dataset. (b) The PBMC68k dataset.

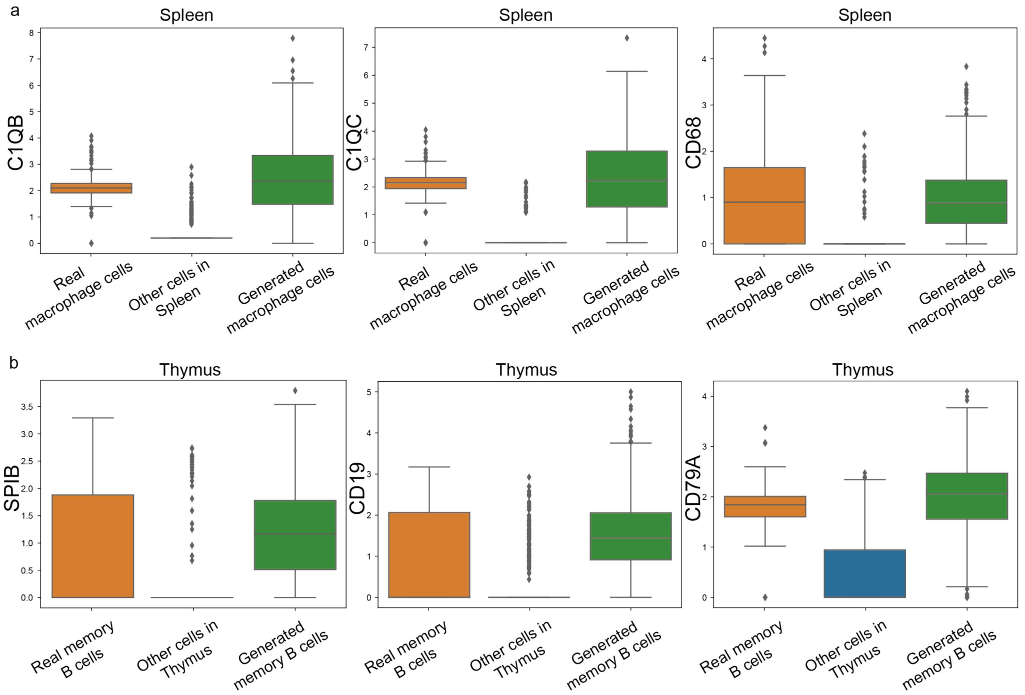

Figure S5: Marker genes' expression levels of real and multi-conditionally generated cells. (a) Maker genes of spleen macrophage cells. (b) Maker genes of thymus memory B cells.

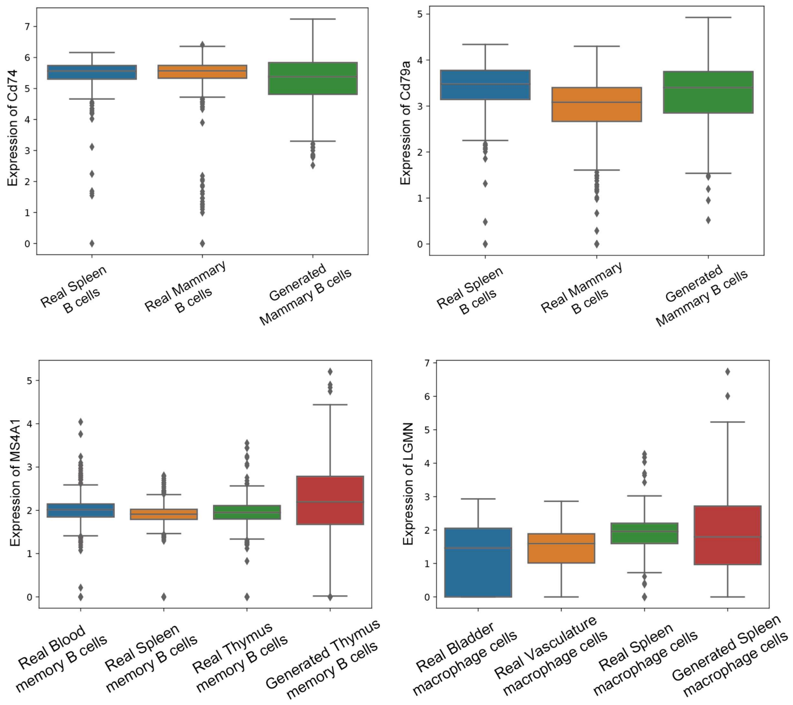

Figure S6: Marker genes' expression levels of real and multi-conditionally generated cells in different organs, showing similar expression levels.

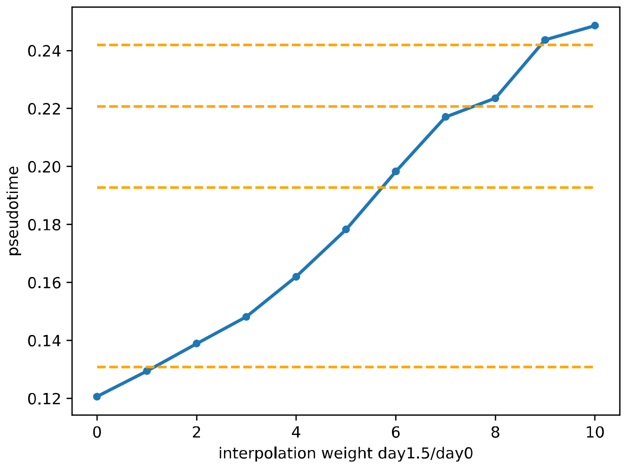

Figure S7: Pseudotime distance of generated states with different interpolation weights. Orange lines are the pseudotime of days 0, 0.5, 1, and 1.5 in the real data, from bottom to up.

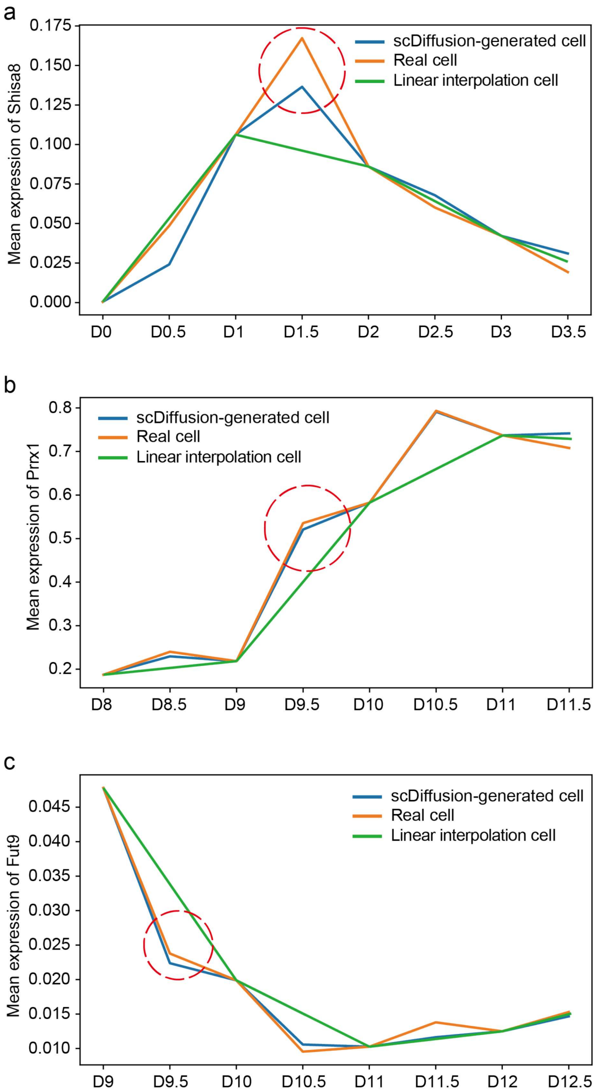

Figure S8: The key TF or marker gene expression in real cells, scDiffusion-generated cells, and linear interpolation-generated cells, respectively. The expression is first normalized and then logarithmized. (a) The expression of *Shisa8*. (b) The expression of *Prrx1*. (c) The expression of *Fut9*.

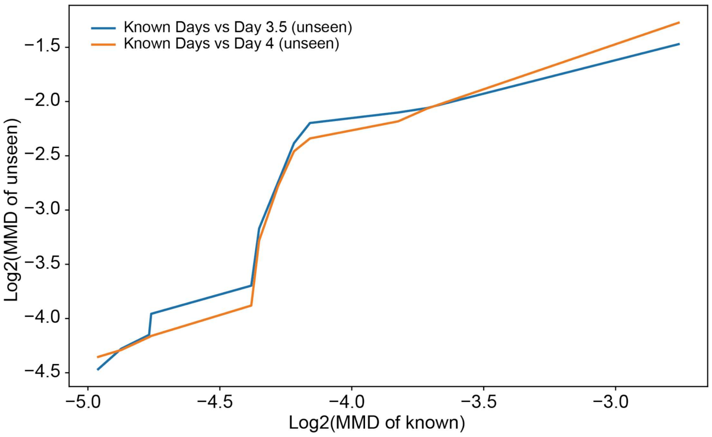

Figure S9: The MMD of known and unseen cells. We generated cells on day 3.5 and day 4 from known cells in the Waddington-OT dataset. We used scDiffusion with autoencoders from different training steps to generate a series of unseen cells and known cells. The MMD between the generated cells and real cells were then calculated. The MMD of generated unseen cells showed positively correlated with the MMD of generated known cells, suggesting that it is possible to estimate the confidence of unseen cells by the results of known cells.
